# Immediate and Long-Term Effects of Breathing Exercises on Reaction Time

**DOI:** 10.3390/medicina60111890

**Published:** 2024-11-18

**Authors:** Burçin Akçay, Ozan Bahadır Türkmen, Ebru Kaya Mutlu, Canan Demir, Ahmet Kurtoğlu, Kopzhassar Bayetov, Madawi H. Alotaibi, Safaa M. Elkholi

**Affiliations:** 1 Department of Physiotherapy and Rehabilitation, Faculty of Health Sciences, Bandırma Onyedi Eylül University, 10250 Balikesir, Türkiye; 2 Department of Coaching Education, Faculty of Sport Science, Bandırma Onyedi Eylül University, 10250 Balikesir, Türkiye; 3 Department of Physical Culture, Faculty of Sports and Arts, Khoja Akhmet Yassawi International Kazakh-Turkish University, Almaty 161200, Kazakhstan; 4 Department of Rehabilitation Sciences, College of Health and Rehabilitation Sciences, Princess Nourah Bint Abdulrahman University, P.O. Box 84428, Riyadh 11671, Saudi Arabia

**Keywords:** breathing exercises, reaction time, choice reaction time, auditory reaction time, visual reaction time

## Abstract

*Background and Objectives*: The aim of this study was to investigate the immediate and long-term effects of breathing exercises on simple and choice reaction time. *Materials and Methods*: A total of 31 participants were included in the study. The participants were randomly divided into two groups. The intervention group (IG; n:16) received 12 sessions of breathing exercises for four weeks (three days a week), while the control group (CG; n:15) received no intervention. Both groups underwent the same assessments at the same times: at baseline, after the first exercise session, and after four weeks/after 12 exercise sessions. Reaction time was assessed using three conditions: choice reaction time (CRT) of the upper limb, auditory reaction time (ART), and visual reaction time (VRT) tests. The first assessment was applied at baseline. The second assessment measured the short-term effect of the breathing exercises after the study group received their first breathing exercise session. The third assessment, to determine the long-term effect, was repeated four weeks after the baseline (A4W) assessment after completing 12 breathing exercises. *Results*: There was no significant difference between the groups at baseline, immediately, and A4W (*p* < 0.05) for the VRT, ART, and CRT results with the dominant hand (DH), whereas a group-by-time interaction was found only for the visual reaction time results with the non-dominant hand (nDH) (*p* > 0.05). *Conclusions*: The results of this study, which included the evaluation of the immediate and long-term effects of breathing exercises on reaction time, showed an improvement in visual reaction time between the groups over time. In further studies, it is recommended to evaluate the changes and responses in the central nervous system with objective methods to reveal the effect of breathing exercises on reaction time more clearly.

## 1. Introduction

Breathing exercise, also known as “breathing”, or “diaphragmatic breathing”, is described as an effective integrative body-mind training for coping with stress and psychosomatic conditions [1,2]. Breathing exercise is an important part of a comprehensive pulmonary rehabilitation program [3,4], but it is also becoming more common in healthy individuals [5,6,7,8].

Several breathing exercises such as slow and deep breathing, relaxation breathing, pursed lip breathing, and diaphragmatic breathing are described in evidence based-physiotherapeutic approaches in pulmonary rehabilitation programs. The effects of these breathing exercises in pulmonary rehabilitation are indicated to be effective in reducing pulmonary hyperventilation and improving respiratory muscle function, exercise tolerance, and quality of life [3]. It is also emphasized that breathing exercises have effects on heart rate, thoracoabdominal movement, respiratory muscle activity, postural stability, and the autonomic nervous system in healthy individuals [1,2,5,6,7,8].

Also, studies on the physiological mechanism of diaphragmatic breathing exercises shows that even one breathing exercise can reduce blood pressure and heart rate variability [9,10,11] and improve oxygenation [12], respiratory function, abdominal muscle strength [13], and stress [14]. However, there are no studies investigating the effects of breathing exercises, which are used in pulmonary physiotherapy and rehabilitation and defined within its framework, on reaction time.

Reaction time is defined as the time between the onset of the stimulus and the onset of the response. Reaction time estimates the individual’s capacity to inhibit pre-potential motor responses. Reduced reaction time means greater alertness, faster information processing, and fewer distractions [15]. Reaction time varies depending on many factors such as age, gender, weight, physical fitness, stimulus type, frequency and intensity, and neuromuscular diseases [16]. Although it is affected by many factors, studies have shown that therapeutic exercises can improve reaction time in different population groups such as the elderly population, individuals with neurological diseases, and athletes [17,18,19]. However, these therapeutic exercises do not include breathing exercises. In the literature, there are studies on the effect of yoga-based breathing exercise, which is not one of the breathing exercises used in physiotherapeutic approaches and is different as a breathing exercise approach, on reaction time [2,20,21,22,23]. Some of these studies stated that yoga-based breathing exercises decreased reaction time, and one of them did not have any effect on reaction time [20,21,22,23]; therefore, the effects of yoga-based breathing exercise on reaction time in the literature are contradictory.

Although many effects of breathing exercises, which are widely used for by physiotherapists, have been reported and the positive effects of other therapeutic exercises on reaction time have been reported, it is seen that the examination of the effect of breathing exercises on reaction time is an important gap in the literature.

We hypothesized that one-session of breathing exercises consisting of diaphragmatic breathing, pursed lip breathing, and thoracic expansion exercises can improve simple and choice reaction time. Also, we hypothesized that 12 sessions of breathing exercises consisting of diaphragmatic breathing, pursed lip breathing, and thoracic expansion exercises can improve simple and choice reaction time. Therefore, the aim of this study was to investigate the immediate and long-term effects of breathing exercises on simple and choice reaction time.

## 2. Materials and Methods

### 2.1. Study Design

The study was conducted as a randomized, controlled, single-blind study.

### 2.2. Participants

This study involved university students at the Department of Physiotherapy and Rehabilitation between December 2022 and February 2023. Each participant was given written and verbal explanations of the procedures to be carried out and was also asked to sign an informed consent form if they agreed to participate. The study was conducted using the “Helsinki Declaration”.

The inclusion criteria were volunteering to participate in the study, being between the ages of 18 and 30, not having yet taken departmental lectures that included breathing exercises, and not having any problem that would prevent respiratory exercise.

Exclusion criteria were the presence of acute infection or chronic disease of neurologic, psychiatric, orthopedic, cardiologic, rheumatologic, etc. origin, any injury and operation (trauma, surgery, fracture, etc.) in the last six months, regular medication use, and presence of respiratory disease.

The study sample size was calculated as at least 15 in each group with a 95% confidence interval and 80% power by G*Power 3.1.9.7 analysis, using reaction time as the criterion from our outcome measures, and using Kumar et al.’s study as an example [22].

The study included 31 participants aged between 20 and 29 years. The participants were randomly divided into two groups as intervention group [n = 16, (female = 13, male = 3)] and control group [n = 15, (female = 11, male = 4)]. Table 1 shows the demographic characteristics of the participants. Accordingly, there was no significant difference between the two groups in terms of age, height, weight, and body mass index (BMI) (*p* > 0.05).

### 2.3. Randomization and Blinding

**Figure 1:**
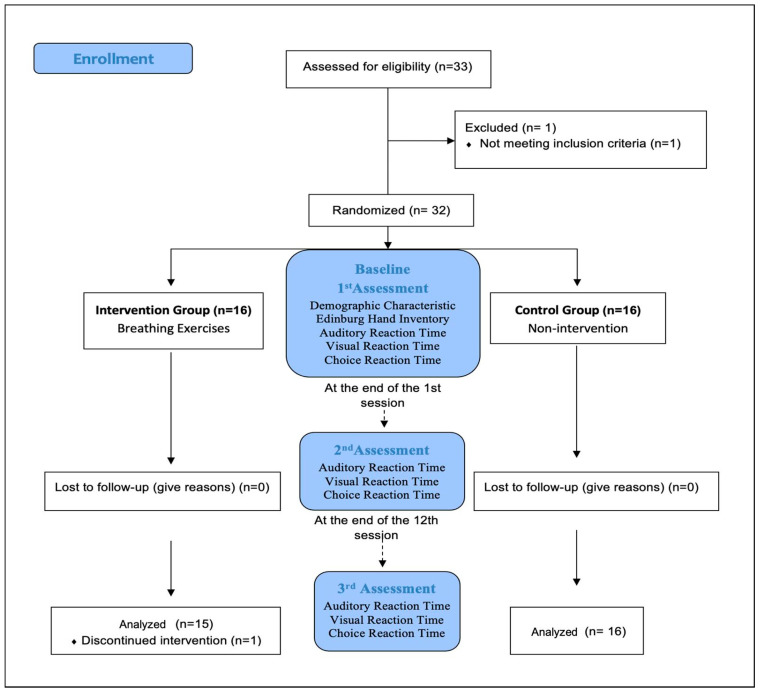
Experimental Design of Study.

Simple randomization (computer-generated random numbers) was used, and sequentially numbered index cards containing the random allocations were prepared by an investigator (a faculty researcher) not clinically involved in the trial to ensure allocation concealment. The physiotherapist (E.K.M.) who delivered the interventions then opened each envelope and allocated participants to the intervention and control groups in accordance with the selected index card. A physiotherapist with 10 years’ experience in cardiopulmonary physiotherapy (C.D.) performed the breathing exercise sessions, while a blinded physiotherapist (B.A., O.B.T.) performed the other assessments and data collection (Figure 1).

### 2.4. Interventions

The intervention group received 12 sessions of breathing exercises for four weeks (three days a week), while the control group received no intervention.

Breathing exercise: During all breathing exercise sessions, participants performed the exercises sitting on a chair. The breathing exercise session started with 5 min of relaxed breathing exercises, followed by 5 min of diaphragmatic breathing, 5 min of pursed lip breathing, and 5 min of thoracic expansion exercises, and then, the session was completed with 5 min of relaxed breathing exercises. So, each breathing exercise session lasted 30 min and included resting time. For the first 5 min and the last 5 min of the session, during the relaxed breathing exercises, the participants were instructed to focus on their breathing and the feelings generated in their bodies while sitting comfortably in chairs with their eyes closed and all limbs relaxed. After 5 min of relaxed breathing, the participants performed a diaphragmatic breathing exercise for 5 min. During the diaphragmatic breathing exercises, they were asked to perform diaphragmatic breathing by contracted diaphragm and inflate the abdominal cavity while inhaling deeply via the nose, after putting the dominant hand on the abdomen and the non-dominant hand on the chest and releasing the diaphragm muscles during exhalation and pulling the abdomen inward while exhaling slowly through the mouth. Five diaphragmatic breaths were performed in 1 min. After 5 min of diaphragmatic breathing, the participant rested for 1 min. They were then requested to perform pursed lip breathing for 5 min. While doing pursed-lip breathing, participants were asked to inflate their whole lungs by breathing deeply through their noses, and then, they were asked to exhale the breath they took for a long time (1:2) and through the mouth. After 5 min of pursed lip breathing in 1 min, they rested for 1 min, and then, thoracic expansion exercises were performed. These exercises were applied for 5 min, and included lower thoracic expansion, lateral costal expansion, and upper chest expansion exercises, with two expansion exercises for each region in 1 min. After completing the thoracic expansion exercises, participants engaged in 5 min of relaxed breathing at the end of the session [24,25] (Table 2).

### 2.5. Outcome Measurements

The baseline assessment was performed after randomization; both groups followed a second assessment after the study group received their first breathing exercise session to measure the immediate effect of respiratory exercises. To determine the long-term impact, the third assessment was repeated four weeks after the baseline assessment after completing 12 sessions of breathing exercises.

Age, gender, weight, height, presence of acute infections and chronic diseases, medications and frequency, exercise habits, mental or sensory problems, upper extremity injuries, and surgical operations in the last six months were recorded as demographic information for the participants.

The Turkish version of the Edinburgh Handedness Inventory determined the dominant hand preference [26,27].

Reaction time is subdivided into simple reaction time, recognition reaction time, choice reaction time, and serial reaction time [15]. In this study, simple reaction time and choice reaction time were examined.

Simple reaction time: In tests where simple reaction time is measured, there is only one stimulus and one response [15]. In this study, simple reaction time was tested in two conditions: auditory and visual reaction time. Visual and auditory reaction tests were evaluated with web-based tests since it was indicated that there was no difference between web-based and laboratory reaction time tests [28]. A computer (MSI VECTOR GP68HX 13VH-208TR model) was used for all simple reaction time tests.

Auditory reaction time (ART) was assessed with a web-based test (playback.fm https://playback.fm/audio-reaction-time/, accessed on 20 December 2022). Sony WH-1000XM4 model headphones were used in this test. Before starting the test, one trial test was conducted. Participants were requested to press the ‘space bar’ key on the computer keyboard when they heard a sound. Five auditory stimuli were presented, and their responses were taken. The average of the five stimulus responses was recorded in milliseconds. The test was repeated three times for dominant and non-dominant hands, and a 1 min rest break was given between repetitions; then, the average of these three repetitions was taken for each hand [29].

The Visual reaction time (VRT) was assessed with a web-based test (https://humanbenchmark.com/tests/reaction-time, accessed on 20 December 2022). Before starting the assessment, one trial test was conducted, and the test was explained. During the test, the participants were asked to place their index finger on the left button of a computer mouse (Logitech G502 X LIGHTSPEED model) and press the left button as soon as the green color lit up on the screen. The time between the light stimulus and the participant’s response was recorded in milliseconds. Each test was administered five times, and the average was recorded. The test was repeated three times for dominant and non-dominant hands, and a 1 min rest break was given between repetitions; then, the average of these three repetitions was taken for each hand [30].

Choice reaction time (CRT): In choice reaction time tests, the user’s response to random stimuli is evaluated [15]. The validated BlazePod sensor was used to test the overall choice reaction time of the upper limb in this study [31]. The test was monitored via Blazepod’s phone application, and outcome measurements were taken. In the Choice reaction test, four BlazePod sensors were used. In each set, 10 blue lights were randomly lit on one of the four sensors. Participants were requested to touch the lighted sensor on the panel as quickly as possible. The BlazePod sensors recorded the time in milliseconds between the light turning on and the participant touching the sensor for each stimulus, and an average of 10 stimuli was given. With a 1 min rest interval, the average of three applications in this design was calculated. A choice reaction test was performed with the same protocol for the dominant and non-dominant sides [32].

### 2.6. Statistical Analysis

The Statistical Package for the Social Sciences 22.0 (SPSS Inc., Chicago, IL, USA) was used for all statistical analyses. In the study, the analyses were visualized with GraphPad Prism 8 (Graphpad Software 8.0.2, Chicago, IL, USA) application and analyses were performed on the mean (m) since the data were normally distributed. Before the statistical analysis, the Kolmogorov–Smirnov test was used to assess the distribution of data. The data were found to be normally distributed, so a parametric test was used for the statistical analysis. Levene’s test was applied for homogeneity of variances in our study. Accordingly, the variances were within acceptable limits. Demographic and clinical baseline variables comparisons of the two groups were conducted using a chi-square analysis for the categorical variables and independent *t*-tests for the continuous variables. Repeated measures analysis of variance (rANOVA) was conducted with time (baseline, immediate, and after four weeks) as a within-subject variable and group (intervention and control group) as a between-subjects variable to analyze the effect of the interventions on the auditory reaction time, visual reaction time, and choice reaction time. In our study, SD values were taken into consideration, and homogeneity of variance was found to be out of limits in some parameters. For this, Mauchly’s Sphericity test results were analyzed and Greenhouse–Geisser correction was applied. Partial eta squared was used as an effect size indicator, elucidated as small, 0.01; medium, 0.06; and large, 0.14 [33]. Secondly, mixed-model repeated-measure analysis of covariance (ANCOVA) was used, with smoking and exercising situations as covariates, to test the effect of breathing exercises on the auditory reaction time, visual reaction time, and choice reaction time at each time interval (baseline, immediate, and after four weeks) as the within-subject variable and the group (intervention and control group) as the between-subjects variable. The significance level was set at *p* < 0.05.

## 3. Results

**Figure 2:**
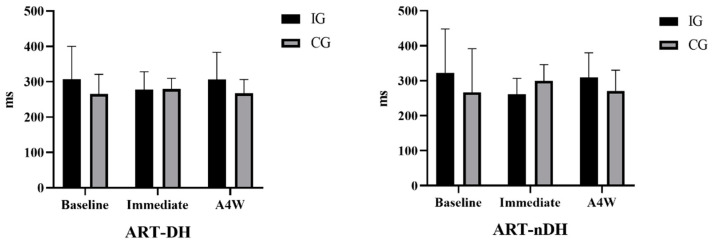
Comparison of ART results of participants. IG: intervention group, CG: control group, ART-DH: auditory reaction time—dominant hand, ART-nDH: auditory reaction time—non-dominant hand, A4W: after 4 weeks.

Figure 2 compares the baseline, immediate, and A4W ART results of the participants’ DH and nDH. Accordingly, ART-DH results in the IG and CG groups were compared at baseline (respectively; m ± sd = 307.19 ± 93.21 ms: 265.60 ± 55.32 ms), immediately (respectively; m ± sd = 277.28 ± 51.13 ms; 279.84 ± 29.84 ms), and A4W (respectively; m ± sd = 306.56 ± 76.48; 267.58 ± 38.82) and no difference was detected in the time [F(1, 29) = 0.199, ηp2 = 0.007, *p* = 0.768] or group*time interaction [F(1, 29) = 1.364, ηp2 = 0.045, *p* = 0.263]. Accordingly, the ART-nDH results in the IG and CG groups were analyzed at baseline (respectively; m ± sd = 322.84 ± 125.38 ms: 266.40 ± 125.16 ms), immediately (m ± sd = 260.86 ± 45.88 ms; 299.92 ± 46. 26 ms) and A4W (respectively; m ± sd = 308.87 ± 71.10; 271.10 ± 59.06) and no difference was detected in time [F(1, 29) = 0.220, ηp2 = 0.008, *p* = 0.704] or group*time interaction [F(1, 29) = 2.673, ηp2 = 0.084, *p* = 0.263].

**Figure 3:**
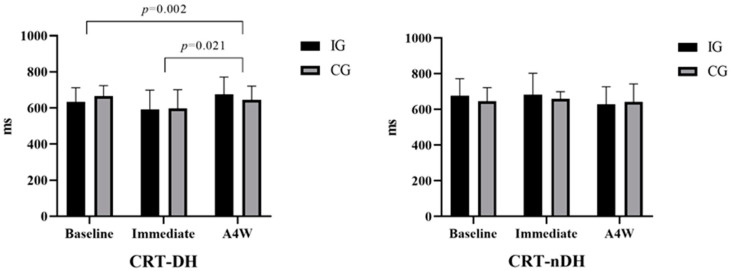
Comparison of CRT results of participants: IG: intervention group, CG: control group, CRT-DH: choice reaction time—dominant hand, CRT-nDH: choice reaction time—non-dominant hand, A4W: after 4 weeks.

Figure 3 compares the baseline, immediate, and A4W CRT results of the participants’ DH and nDH. Accordingly, CRT-DH results in IG and CG groups were compared at baseline (respectively; m ± sd = 690.41 ± 86.59 ms: 651.46 ± 70.68 ms), immediately (respectively; m ± sd = 633.24 ± 79.05 ms; 665.60 ± 58.41 ms), and A4W (respectively; m ± sd = 592.75 ± 106.18; 597.93 ± 103.54). According to these results, there was a significant difference in time interaction [F(1, 29) = 8.703, ηp2 = 0.231 (large effect), *p* < 0.001]. In the posthoc test (Bonferroni), there was a significant difference between participants’ baseline (m ± sd = 670.93 ± 14.25) and A4W (m ± sd = 595.34 ± 18.85) results [mean difference = 75.59, std. err = 19.69, *p* = 0.002 (95% CI = 25.5 to 125. 6)] and there was a significant difference between immediate (m ± sd = 649.42 ± 12.55 ms) and A4W (m ± sd = 595.34 ± 18.85 ms) results [mean difference = 54.08, std. err = 18.63, *p* = 0.021 (95% CI = 6.7 to 101.4)]. But there was no difference group*time interaction [F(1, 29) = 1.857, ηp2 = 0.06, *p* = 0.165]. Accordingly, CRT-nDH results in IG and CG were analyzed at baseline (respectively; m ± sd = 675.44 ± 96.14 ms: 645.73 ± 75.84 ms), immediate (respectively; m ± sd = 681.32 ± 120.8 ms; 658.31 ± 40.03 ms) and A4W (respectively; m ± sd = 628.25 ± 97.67 ms; 640.93 ± 101.05 ms). There was no significant difference in time [F(1, 29) = 1.675, ηp2 = 0.055, *p* = 0.196] and group*time interaction [F(1, 29) = 0.652, ηp2 = 0.022, *p* = 0.525].

**Figure 4:**
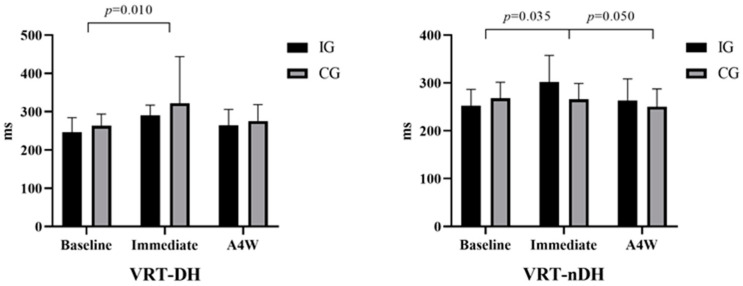
Comparison of VRT results of participants: IG: intervention group, CG: control group, VRT-DH: visual reaction time—dominant hand, VRT-nDH: visual reaction time—non-dominant hand, A4W: after 4 weeks.

Figure 4 compares the baseline, immediate, and A4W VRT results of the participants’ DH and nDH. Accordingly, the VRT-DH results in the IG and CG groups were compared at baseline (respectively; m ± sd = 246.37 ± 38.06 ms: 265.16 ± 30.63 ms), immediately (respectively; m ± sd = 290.60 ± 26.85 ms; 321.7 ± 122.21 ms), and A4W (respectively; m ± sd = 264.43 ± 41.44; 275.79 ± 42.70). According to these results, there was a significant difference in time interaction [F(1, 29) = 6.907, ηp2 = 0.192 (large effect), *p* = 0.005]. In the posthoc test (Bonferroni), there was a significant difference between participants’ baseline (m ± sd = 254.77 ± 6.23) and immediate (m ± sd = 306.15 ± 15.64) results [mean difference = −15.34, std. err = 9.45, *p* = 0.010 (95% CI = −39.3 to 8.6)]. However, there was no difference group*time interaction [F(1, 29) = 0.258, ηp2 = 0.009, *p* = 0.713]. Accordingly, VRT-nDH results in the IG and CG groups were analyzed at baseline (respectively; m ± sd = 252.25 ± 34.36 ms: 267.71 ± 33.67 ms), immediately (respectively; m ± sd = 301.71 ± 55.89 ms; 265.99 ± 32.91 ms), and A4W (respectively; m ± sd = 263.31 ± 45.18 ms; 250.12 ± 37.34 ms). There was a significant difference in time [F(1, 29) = 4.992, ηp2 = 0.147 (large effect), *p* = 0.010] and group*time interaction [F(1, 29) = 3.744, ηp2 = 0.114 (large effect), *p* = 0.030]. Accordingly, there was a significant difference in the IG group between the at-baseline (m ± sd = 259.98 ± 6.11) and immediate (m ± sd = 283.85 ± 8.31) results [mean difference = −23.87, std. err = 8.88, *p* = 0.035 (95% CI = −46.4 to −1.29)]. There was also a significant difference between the immediate (m ± sd = 283.85 ± 8.31) and A4W (m ± sd = 256.72 ± 7.47) results [mean difference = 27.13, std. err = 10.66, *p* = 0.050 (95% CI = 0.03 to 54.2)].

## 4. Discussion

The present study aimed to evaluate the immediate and long-term effects of breathing exercises on reaction time and was conducted in a randomized, controlled, single-blind trial. While 12 sessions of breathing exercises were performed for four weeks (three days a week) in the intervention group, no intervention was applied to the control group. As a result of this study, significant changes were demonstrated over time in the choice reaction time using the dominant hand and in the visual reaction time using the non-dominant hand. However, only the visual reaction time using the non-dominant hand was observed to show a significant change in the group–time interaction.

In the literature, although breathing exercises including diaphragmatic breathing, pursed lip breathing, and thoracic expansion exercises are widely reported to be effective in many fields in physiotherapy [5,6,7,8], there is no study investigating the short-term or immediate effect on reaction time. In the present study, no change was observed in the auditory or choice reaction time after one session of breathing exercises, whereas a statistically significant increase in visual reaction time with the non-dominant hand was detected. However, unlike the breathing exercises we used in this study, there are studies that examined the relationship between yoga-based breathing exercises and reaction time. The first of these studies, by Kumar et al., examined the immediate effect on reaction time after breathing slowly and deeply through one nostril at six cycles per minute in the elderly. As a result, it was reported that right-nostril breathing decreased auditory reaction time and visual reaction time, while left-nostril breathing increased the reaction time [22]. In another study by Bhavanani et al., 22 healthy school-age children showed a decrease in auditory and visual reaction time after nine repetitions of yogic breathing exercises defined as mukh bhastrika [20]. In addition, in this study, the immediate effect at the end of a 30 min session was evaluated, and this duration was longer than that of the other studies [20,22]. In addition, reaction time varies depending on age [16]. Therefore, in the present study, young adults between the ages of 18 and 30 were included, and no significant difference was found between the groups in mean age. Studies have reported an acute effect of aerobic exercise on reaction time and suggested that this effect is due to changes in the neurotransmitter mechanisms of the brain caused by aerobic exercise [34,35]. Nevertheless, since the effect of breathing exercises is different from aerobic exercises, more studies are needed to investigate the reasons for the effect on visual reaction time but not on auditory and choice reaction time and to examine the dose effect of exercise with a more detailed analysis.

In the current study, compared to the control group, 12 sessions of breathing exercises demonstrated no significant difference in choice or auditory reaction time, but there was a significant decrease in visual reaction time. Although there are studies in the literature examining the effects of yoga-based breathing exercises on reaction time in the long term, there are no studies investigating the effects of breathing exercises such as diaphragmatic breathing, pursed lip breathing, and thoracic expansion exercise similar to the present study. Considering the studies investigating yoga-based breathing exercises, Shelke et al. [36] reported a decrease in visual reaction time after three months of yoga-based breathing exercises, Doiphode et al. [37] reported a decrease in auditory and visual reaction time after a six-week intervention, and Telles et al. [27] reported no significant change in visual reaction time after 12 months of intervention. In all of these studies, only yoga-based breathing exercises were used, without the use of yoga posture exercises [27,28,29,30,31,32,33,34,35,36,37]. Therefore, these are used to compare the results of our study. Although breathing exercises were applied differently from previous studies [23,36,37], similar results were found in the study of Shelke et al. [36]. However, since the effect of breathing exercises applied for four weeks was evaluated in this study, this period was even shorter than in previous studies. In future studies, the measurement of reaction time after a longer period of exercise is recommended.

In the literature, it has been reported that diaphragmatic breathing exercises activate and increase parasympathetic tone [1,2,24]. Since the protocol applied in this study was a similar breathing exercise protocol to that of Soer et al. [24], the improvement in visual reaction time may be due to the fact that breathing exercises increase the activation of the parasympathetic system, similar to this study. However, to present the mechanism of these effects, it is recommended that future studies measure the findings of physiological autonomic system activity after each exercise session and after the completion of all interventions. However, it is still conceivable that breathing exercises may positively improve reaction time, especially in visual reaction time. Also, investigating the effects of breathing exercises on reaction time in individuals with respiratory diseases or diseases affecting reaction time in comparison with the control group may more precisely clarify the relationship between breathing exercises and reaction time.

### Limitation

The primary limitation is that there were mostly female participants in the groups, and the effect of the reaction time on women’s hormonal levels was not measured in this study. The other limitation is that people’s stress levels were not evaluated. If the physiologic findings of the autonomic nervous system had been evaluated at baseline and the end of each breathing exercise session, the causes and relationships of the effects on reaction time could have been more clearly demonstrated. Reaction time is also affected by age and gender, but this study included only young adults. Further studies are recommended to investigate gender differences and changes between age groups. Although the properties of the computer used in this study were favorable, factors such as internet connection, which may affect the latency of the computer and, thus, the accuracy of the measurement, were not standardized. In future studies, it is recommended that additional technical and software equipment be used to reduce the error in reaction time measurements where milliseconds are important.

The strengths of this study were that it was designed as a randomized, controlled, single-blind trial and that all reaction time tests were performed with the dominant and non-dominant hands. Another strength is that this is the first study in the literature to examine the effect of breathing exercises such as diaphragmatic breathing, pursed lip breathing, and thoracic expansion on reaction time. This study was conducted in healthy individuals and young adults. However, it is a pioneer for further studies to investigate the effects of breathing exercises on reaction time in different disease and age groups.

## 5. Conclusions

The results of this study, which included an assessment of the immediate and long-term effects of breathing exercises on reaction time, showed an improvement in visual reaction time between groups over time. The immediate effects of breathing exercises were found to have no effect on auditory and choice reaction time but affected visual reaction time. After four weeks of breathing exercise intervention, only an improvement in visual reaction time was observed. Immediate and long-term breathing exercises can improve visual reaction time parameters. Therefore, breathing exercises may be effective in individuals whose reaction time is affected by aging or any disease. In future studies, it is recommended that the changes and responses in the central nervous system be evaluated with objective methods to reveal the effect of breathing exercises on reaction time more clearly. Also, it is recommended to apply the study in expanded numbers of participants using different data collection tools.

### Clinical Implication and Future Directions

Breathing exercises can be an effective and applicable method in some conditions where reaction time decreases due to various diseases or aging. Therefore, the effect of breathing exercises on reaction time in different disease groups, ages, and genders can be examined in further studies. Also, to demonstrate the effect of breathing exercises on reaction time more clearly, it is recommended to evaluate the changes and responses of the central nervous system with more objective methods such as electroencephalography.

## Figures and Tables

**Table 1:** Baseline demographics features of groups.

	IG	CG	*p*
Age (Years)	21.18 ± 2.19	20.93 ± 0.96	0.683
Height (cm)	167.378 ± 7.34	170.738 ± 10.13	0.297
Weight (kg)	61.068 ± 10.44	69.568 ± 19.22	0.131
BMI (kg/m^2^)	21.75 ± 3.24	22.53 ± 3.29	0.240

IG = Intervention Group, CG = Control Group, BMI = Body Mass Index.

**Table 2:** The breathing exercise session.

Duration	Exercise	Instructions/Details
0–5 min	Relaxed breathing exercise	Participants focus on their breathing with eyes closed, sitting comfortably on a chair with all joints relaxed.
5–10 min	Diaphragmatic breathing exercise	The dominant hand is placed on the abdomen and the other hand on the chest. A deep breath is taken through the nose, inflating the abdomen, and slowly exhaled through the mouth, drawing the abdomen inward. Five breaths per minute.
10–11 min	Rest	Participants rest for 1 min.
11–16 min	Pursed lip breathing exercise	Deep breath through the nose; exhale slowly through the mouth for twice as long (1:2 ratio).
16–17 min	Rest	Participants rest for 1 min.
17–24 min	Thoracic expansion exercises	Exercises for upper chest, lateral costal, and lower thoracic expansion. Two expansion exercises per region.
24–25 min	Rest	Participants rest for 1 min.
25–30 min	Relaxed breathing exercise	The session ends with 5 min of relaxed breathing, eyes closed and all joints relaxed.

## Data Availability

The data presented in this study are available on request from the corresponding author.
